# Initial CPAP of 5 versus 8 cmH_2_O during delivery room stabilisation of very preterm infants: the PHILODENDROOM pilot randomised trial

**DOI:** 10.1007/s00431-026-07322-6

**Published:** 2026-08-17

**Authors:** Francesco Cavigioli, Ilia Bresesti, Francesca Castoldi, Sara Gatto, Enrica Lupo, Samantha Rossi, Azzurra La Verde, Silvia Bianchi, Fabio Meneghin, Francesca Viaroli, Valentina Pivetti, Valeria Manfredini, Giulia Cannata, Petrina Bastrenta, Paola Fontana, Ilaria Stucchi, Irene Daniele, Marco Chiera, Gianluca Lista

**Affiliations:** 1 NICU “V. Buzzi” Children’s Hospital, ASST FBF-Sacco, Milan, Italy; 2 Department of Medicine and Surgery, University of Insubria, Varese, Italy; 3 Foundation Centre, Osteopathic Medicine (COME) Collaboration, 65121 Pescara, Italy

**Keywords:** Preterm infants, Delivery room, Neonatal resuscitation, CPAP levels

## Abstract

The objective was to evaluate the feasibility and safety of conducting a randomised controlled trial comparing two initial continuous positive airway pressure (CPAP) levels during delivery room (DR) stabilisation of very preterm infants at risk for respiratory distress syndrome (RDS) and to explore their early physiological respiratory effects. In this pilot randomised controlled trial conducted at a tertiary neonatal intensive care unit, preterm infants born between 26 + 0 and 29 + 6 weeks’ gestation requiring respiratory support at birth were randomised to receive CPAP at either 5 cmH_2_O or 8 cmH_2_O via face mask and T-piece resuscitator during the first 15 minutes of life. The primary objective was to assess trial feasibility and safety. Pre-specified exploratory outcomes included the preductal SpO_2_/FiO_2_ (S/F) ratio at 15 minutes after birth, need for positive pressure ventilation (PPV), surfactant administration, respiratory support requirements and respiratory morbidity. Fifty-six infants were enrolled and equally randomised between groups. The pre-defined sample size was achieved, protocol adherence was completed, and no participant was lost to follow-up, confirming the feasibility of trial conduct. Baseline characteristics were comparable. No significant differences were observed in pre-defined safety outcomes, including pneumothorax, pulmonary interstitial emphysema, or DR intubation. The S/F ratio at 15 min was similar between groups (271 ± 101 vs. 269 ± 111; *p* = 0.95). The proportion of infants requiring PPV was identical in both groups (35%). Although the overall surfactant administration rate did not differ, infants receiving 5 cmH_2_O reached surfactant treatment threshold earlier than those receiving 8 cmH_2_O (2.2 ± 1.7 vs 3.5 ± 2.1 h; *p* = 0.03). Trends towards shorter MV duration and lower BPD incidence were observed in the 8 cmH_2_O group, without statistical significance.

*Conclusion*: This pilot randomised trial demonstrated that comparing two initial CPAP strategies during DR stabilisation of very preterm infants is feasible and no significant adverse events have been reported. While higher initial CPAP was associated with delayed achievement of the FiO_2_ threshold for surfactant administration, exploratory physiological and clinical findings should be considered hypothesis-generating. Adequately powered multicentre trials incorporating physiological bedside assessment of lung aeration are needed to determine the optimal initial CPAP strategy during neonatal transition.

**Table:** 

**What is Known:** • *CPAP is a cornerstone of respiratory support for very preterm infants in the DR, but the optimal initial distending pressure remains uncertain.* • *Higher CPAP levels may improve lung recruitment, although concerns persist regarding air leaks and potential harm.*	
**What is New:** • *In this pilot trial, both 5 and 8 cmH2O initial CPAP levels were feasible and safe during DR stabilisation of very preterm infants.* • *Higher initial CPAP was associated with delayed surfactant threshold achievement, highlighting the potential role of additional tools such as lung ultrasound to tailor and guide individualised respiratory support.*

**What is Known:**

• *CPAP is a cornerstone of respiratory support for very preterm infants in the DR, but the optimal initial distending pressure remains uncertain.*

• *Higher CPAP levels may improve lung recruitment, although concerns persist regarding air leaks and potential harm.*

**What is New:**

• *In this pilot trial, both 5 and 8 cmH2O initial CPAP levels were feasible and safe during DR stabilisation of very preterm infants.*

• *Higher initial CPAP was associated with delayed surfactant threshold achievement, highlighting the potential role of additional tools such as lung ultrasound to tailor and guide individualised respiratory support.*

## Introduction

Preterm infants, particularly those born at less than 32 weeks’ gestation, face significant risks of respiratory distress syndrome (RDS), starting immediately after birth. This condition is mainly characterised by surfactant deficiency and immature lung development. The prevalence of RDS among preterm infants remains a leading cause of neonatal morbidity and mortality, needing timely and effective interventions to ensure optimal short- and long-term outcomes [1, 2].

Immediate and appropriate respiratory support in the delivery room (DR) is critical to allow the clearance of lung fluid and to reach an early and adequate functional residual capacity (FRC), thereby mitigating complications such as atelectasis or overinflation, hypoxaemia and subsequent lung injury. The establishment of an adequate FRC during the first minutes after birth is one of the key determinants of successful neonatal transition, particularly in surfactant-deficient preterm lungs. A well-known strategy involves the use of continuous positive airway pressure (CPAP) [3], which has been shown to provide continuous distending pressure that keeps the alveoli open during expiration, reducing the work of breathing and the need for mechanical ventilation (MV).

Advances in neonatal care have made CPAP a cornerstone of non-invasive respiratory support, yet the interplay between initial CPAP levels and subsequent respiratory outcomes remains a critical area for research. While higher CPAP may promote better alveolar recruitment, concerns are raised about potential adverse effects, such as air leak syndromes and haemodynamic impairment [4]. Therefore, understanding the balance between these effects is essential to refining clinical practice and improving the short- and long-term outcomes for this vulnerable population.

Current European Consensus Guidelines recommend initiating CPAP at approximately 6 cmH_2_O in spontaneously breathing preterm infants, with subsequent titration according to clinical response. However, this recommendation is largely based on limited evidence, and the optimal initial distending pressure during DR stabilisation remains uncertain [5].

To address the current lack of evidence regarding the optimal initial CPAP level during DR stabilisation, we conducted a pilot randomised controlled trial (RCT) comparing 5 cmH_2_O versus 8 cmH_2_O in very preterm infants.

## Methods

### Study design

This investigator-initiated pilot RCT was conducted at the level 3 neonatal intensive care unit of “V. Buzzi” Children’s Hospital, Milan, Italy, between April 2020 and November 2023. The primary objective of the study was to evaluate the feasibility and safety of conducting a future multicentre RCT comparing two initial continuous positive airway pressure (CPAP) strategies during DR stabilisation of very preterm infants. Secondary exploratory objectives were to compare early physiological respiratory adaptation and short-term respiratory outcomes between the two treatment groups.

The study was approved by the Ethics Committee Milano Area 1 (approval number 2018/ST/231), registered at ClinicalTrials.gov (NCT06123845) and conducted in accordance with the Declaration of Helsinki. This manuscript was prepared according to the CONSORT 2010 Statement and the CONSORT extension for randomised pilot and feasibility trials.

#### Study population

Preterm infants born between 26 + 0 and 29 + 6 weeks’ gestation requiring non-invasive respiratory support immediately after birth were eligible for inclusion.

Infants with major congenital malformations, either diagnosed antenatally or postnatally, or whose parents declined participation, were excluded.

Written informed consent was obtained antenatally from the parents or legal guardians before delivery.

#### Randomisation and allocation

Participants were randomly assigned in a 1:1 ratio to receive an initial CPAP level of either 5 cmH_2_O (CPAP 5 group) or 8 cmH_2_O (CPAP 8 group).

The randomisation sequence was generated by an independent investigator using computer-generated random numbers. Sequentially numbered opaque sealed envelopes were prepared before trial commencement. Eligible infants were enrolled by the attending neonatologist, who opened the next envelope immediately before intervention. Owing to the nature of the intervention, blinding of the clinical team was not feasible. However, investigators responsible for outcome assessment and data analysis remained blinded to treatment allocation whenever applicable.

All analyses were performed according to the intention-to-treat principle.

#### Intervention

Respiratory support was initiated immediately after birth using a T-piece resuscitator (Neopuff™, Fisher & Paykel Healthcare, Auckland, New Zealand) connected to appropriately sized face mask. The assigned CPAP level (5 or 8 cmH_2_O) was maintained during the first 15 minutes after birth unless escalation to positive pressure ventilation (PPV) or endotracheal intubation became clinically necessary according to neonatal resuscitation guidelines.

The initial inspired oxygen fraction (FiO_2_) was set at 0.30 and subsequently adjusted to maintain oxygen saturation within the target ranges recommended by contemporary international neonatal resuscitation guidelines. Gas flow was maintained at 10 L/min in all infants.

After the first 15 minutes of life, CPAP was standardised to 6 cmH_2_O in both groups until NICU entry. Following respiratory management was based on local NICU protocols.

Surfactant replacement therapy (Poractant alfa, Curosurf®, Chiesi Farmaceutici, Parma, Italy) was administered at an initial dose of 200 mg/kg when infants reached the recommended FiO_2_ threshold (≥ 0.30) while receiving CPAP, in accordance with the European Consensus Guidelines available during the study period [6, 7].

#### Monitoring during DR stabilisation

Preductal oxygen saturation was continuously monitored using a pulse oximeter sensor placed on the right hand or wrist immediately after birth. Pulse oximetry measurements were obtained using Masimo SET® technology (Masimo Corp., Irvine, CA, USA).

Respiratory support was continuously recorded using a respiratory function monitor (New Life Box Neo-RDS, Advanced Life Diagnostics UG, Germany), allowing simultaneous acquisition of airway pressure, gas flow, tidal volume, oxygen saturation, heart rate and inspired oxygen concentration throughout DR stabilisation. These recordings were subsequently reviewed to verify protocol adherence and accurately document respiratory interventions.

#### Outcomes

The primary objective of this pilot trial was to evaluate the feasibility of conducting a multicentre RCT comparing two initial CPAP strategies.

Because of the exploratory nature of this investigator-initiated pilot study, no formal progression criteria were prespecified. Feasibility was judged pragmatically according to recruitment success, protocol adherence, intervention fidelity and completeness of outcome collection. Importantly, the present study was designed as a pilot trial and was therefore not powered to demonstrate superiority of either CPAP strategy with respect to clinical respiratory outcomes.

Safety was evaluated by monitoring predefined adverse events including pneumothorax, pulmonary interstitial emphysema and endotracheal intubation during the early postnatal period.

Pre-defined exploratory physiological outcomes included preductal oxygen saturation (SpO_2_), inspired oxygen fraction (FiO_2_), heart rate and the preductal SpO_2_/FiO_2_ (S/F) ratio during the first 15 minutes after birth. The S/F ratio was selected as a non-invasive surrogate marker of oxygenation efficiency during neonatal transition and to generate preliminary physiological data for the design of a future definitive trial. Although not a direct measure of lung aeration, changes in the S/F ratio may indirectly reflect the establishment of FRC and effective gas exchange.

Exploratory clinical outcomes included the need for PPV, duration of PPV, surfactant administration, timing of surfactant administration, intubation within the first 72 h of life, duration of mechanical ventilation (MV), bronchopulmonary dysplasia (BPD), mortality and the composite outcome of BPD/death.

#### Sample size

As recommended for pilot randomised trials, the sample size was determined to assess feasibility and obtain preliminary estimates of treatment effects rather than to demonstrate definitive clinical efficacy. No formal hypothesis testing for superiority was planned. Accordingly, the study was not powered to detect statistically significant differences in clinical outcomes, which should therefore be interpreted as exploratory. The planned sample size was calculated according to the confidence interval approach proposed by Cocks and Torgerson [8] Based on this approach, a total sample size of 56 infants (28 per group) was considered appropriate.

#### Statistical analysis

Statistical analysis was performed using the R statistical software, version 3.6.1, within the RStudio development environment, Version 1.4.1103.

Continuous variables are presented as mean ± standard deviation (SD) or median (interquartile range), as appropriate, while categorical variables are presented as counts and percentages. Baseline characteristics were compared using the independent-samples *t*-test or Wilcoxon–Mann–Whitney test for continuous variables and the *χ*^2^ test or Fisher’s exact test for categorical variables, as appropriate.

Repeated measurements of heart rate, preductal oxygen saturation, inspired oxygen fraction and the SpO_2_/FiO_2_ ratio during the first 15 minutes after birth were analysed using a robust mixed-design analysis of variance to evaluate the effects of treatment group, time and treatment-by-time interaction.

As this was a pilot study, all physiological and clinical outcome analyses were prespecified as exploratory analyses. Statistical significance was defined as a two-sided *p*-value < 0.05.

## Results

### Feasibility outcomes

**Fig. 1:**
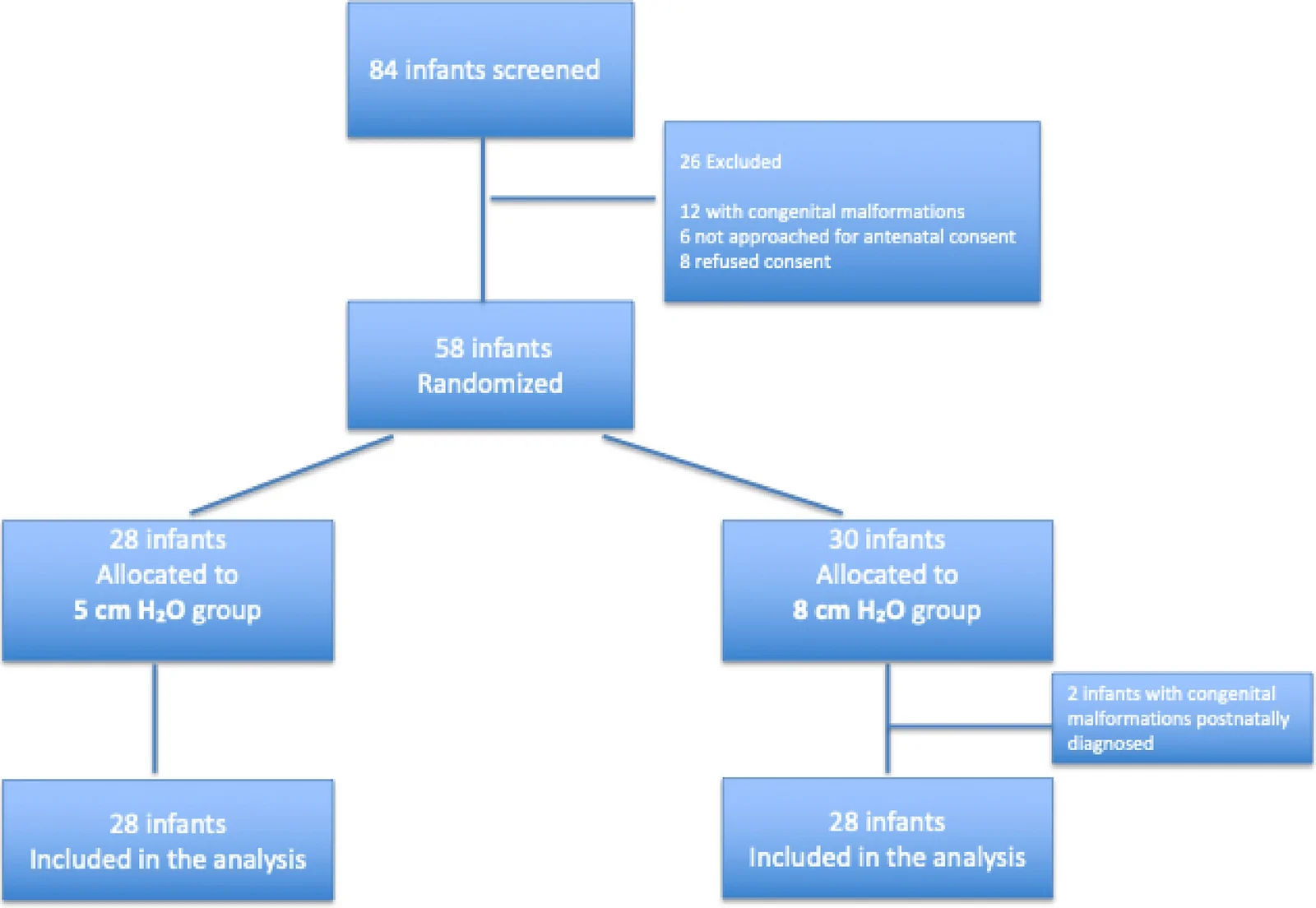
Flow chart of the patients’ selection process

During the study period, 56 very preterm infants were successfully enrolled and randomised, with 28 allocated to each study group (Fig. 1). Recruitment was temporarily slowed by the COVID-19 pandemic; however, the predefined sample size was ultimately achieved.

All randomised infants received the allocated intervention without crossover according to protocol and completed the study. No protocol deviations occurred, and outcome data were available for all enrolled participants, confirming excellent protocol adherence, complete outcome ascertainment and the overall feasibility of the study procedures.

Data are outlined in Table 1.

**Table 1:** Predefined pilot trial objectives to test the feasibility

Outcome	Prespecified	Achieved
Feasibility objective	Prespecified	Achieved
Recruitment target	56 infants	56 (100%)
Allocation adherence	≥ 95%	100%
Intervention fidelity	All infants receive allocated treatment	100%
Missing outcome data	< 5%	0%

### Baseline characteristics

Baseline demographic and perinatal characteristics are summarised in Table 2.

**Table 2:** Main population characteristics

Characteristic	CPAP level 5 (*n* = 28)	CPAP level 8 (*n* = 28)	*p*-value
Male gender, *n (%)*	13 (46.4%)	13 (46.4%)	> 0.99
Singleton, *n (%)*	11 (39.3%)	15 (53.6%)	0.421
Gestational age (weeks), *mean* ± *SD*	28.20 ± 1.10	28.70 ± 1.19	0.112
Birth weight (g), *mean* ± *SD*	1038.8 ± 238.5	1185.9 ± 308.2	0.051
SGA, *n (%)*	3 (10.7%)	2 (7.4%)	> 0.99
Prenatal steroids, *n (%)*	25 (89.3%)	24 (88.9%)	> 0.99
pPROM, *n (%)*	7 (25.0%)	3 (10.7%)	0.491
Apgar score at 5 min, *median [IQR]*	8 [8–9]	8 [8–9]	0.493
Cord lactate (mmol/L), *mean* ± *SD*	3.18 ± 1.34	2.30 ± 0.67	0.011

The two groups were well balanced with respect to gestational age, birth weight, gender, Apgar scores and other relevant perinatal characteristics.

### Safety outcomes

Both interventions were well tolerated. No statistically significant differences were observed in pre-defined safety outcomes between the two groups. Pneumothorax occurred in 0/28 infants in the CPAP 5 group and 3/28 infants in the CPAP 8 group (*p* = 0.23). Pulmonary interstitial emphysema occurred in 0/28 versus 2/28 infants, respectively (*p* = 0.49). Delivery room intubation was uncommon (2/28 vs 1/28, *p* = 0.6).

### Exploratory physiological outcomes

**Fig. 2:**
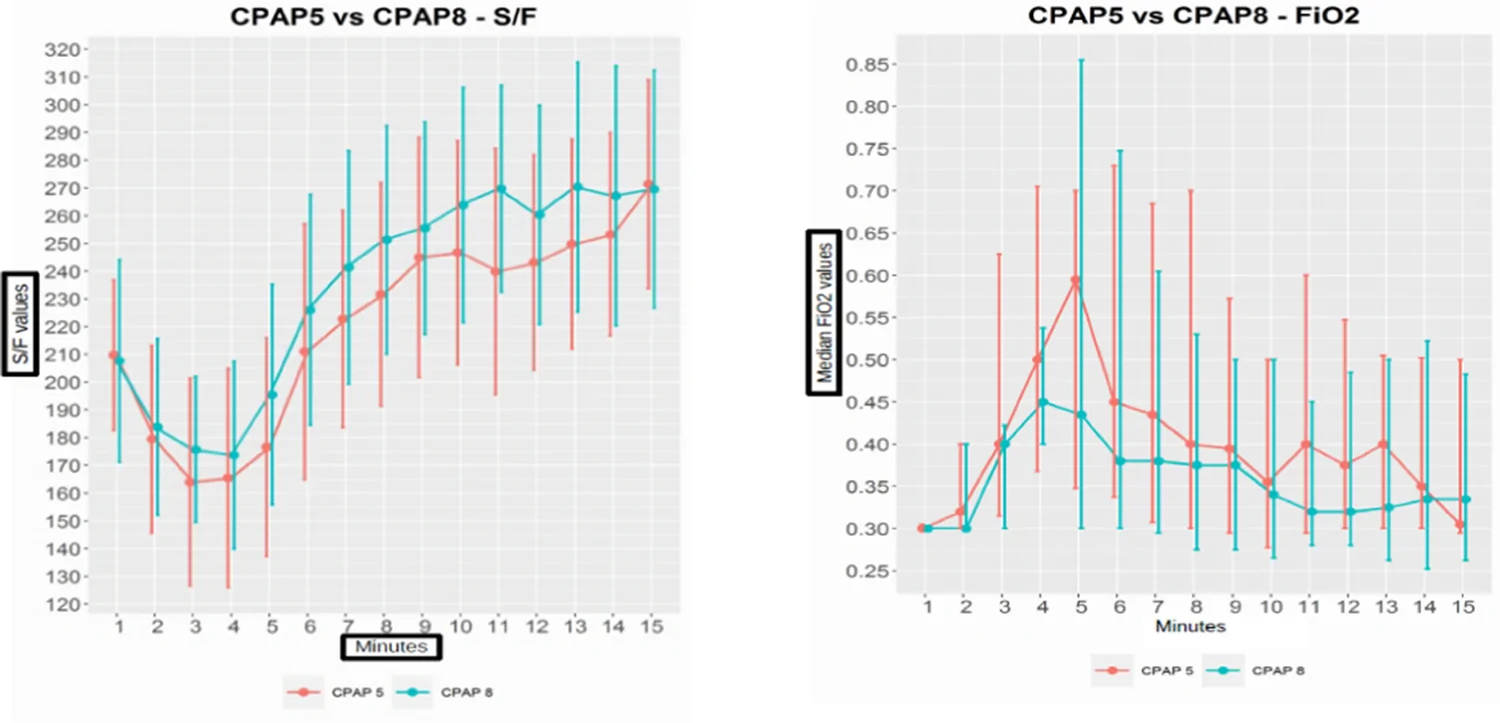
The mean S/F ratio in the first 15 min of life in the two groups

The preductal SpO_2_/FiO_2_ ratio during the first 15 min after birth was similar in the two treatment groups (Fig. 2). At 15 min, the S/F ratio was 271 ± 101 in the CPAP 5 group and 269 ± 111 in the CPAP 8 group (*p* = 0.950). Likewise, FiO_2_ requirements and oxygen saturation trends over time did not differ significantly between groups (Fig. 2).

Ten infants (35.7%) in each group required positive pressure ventilation during DR stabilisation (*p* = 1.0). The duration of PPV was shorter in the CPAP 8 group, although the difference was not statistically significant (51 vs. 85 s, *p* = 0.200).

The overall proportion of infants receiving surfactant during the NICU stay was similar between groups (22/28 vs. 20/28, *p* = 0.900). However, infants allocated to CPAP 5 reached the pre-defined surfactant treatment threshold significantly earlier than those receiving CPAP 8 (2.2 ± 1.7 vs. 3.5 ± 2.1 h, *p* = 0.030).

Intubation rate in the first 72 h after birth was 9/28 vs 7/28 in the two groups (*p* = 0.56). Duration of MV, BPD and the composite outcome of BPD or death did not differ significantly between groups, although a trend toward shorter MV and lower BPD rates was observed in infants initially treated with CPAP 8. The rates of combined BPD or death were comparable.

## Discussion

In this pilot RCT, we demonstrated that comparing two different initial CPAP strategies during DR stabilisation of very preterm infants is feasible. To our knowledge, this is among the first RCTs specifically comparing two fixed initial CPAP levels during the initial fetal to neonatal transition of very preterm infants, while combining standardised respiratory management with continuous physiological monitoring using a respiratory function monitor. Although not designed to demonstrate treatment efficacy, this study provides important feasibility data together with preliminary physiological observations that may shape the design of future multicentre trials evaluating individualised respiratory support strategies. Even if exploratory physiological outcomes did not demonstrate significant differences in oxygenation during the first minutes after birth, higher initial CPAP was associated with delayed achievement of the FiO_2_ threshold for surfactant administration without evidence of increased short-term respiratory complications. These findings should be considered hypothesis-generating and provide important preliminary data to design future studies.

One of the main objectives of this study was to evaluate whether a randomised comparison of two initial CPAP strategies could be successfully implemented in the DR. Despite the organisational challenges imposed by the COVID-19 pandemic, recruitment targets were achieved, protocol adherence was excellent and no participant was lost to follow-up. These findings support the feasibility of conducting a larger multicentre randomised trial evaluating DR CPAP strategies.

In the present pilot study, higher initial CPAP was not associated with an observed increase in predefined respiratory complications such as pneumothorax or pulmonary interstitial emphysema. Although promising, the study was not powered to detect differences in adverse events, and these favourable findings should be interpreted cautiously.

The optimal level of distending pressure during DR stabilisation remains uncertain, despite CPAP being the cornerstone of respiratory support for spontaneously breathing preterm infants. Therefore, identifying the most appropriate strategy to promote lung aeration while minimising respiratory injury remains an important clinical challenge.

At the time this study was designed, initial CPAP levels of 5 cmH_2_O were still widely adopted in clinical practice, whereas emerging physiological and experimental evidence suggested that higher distending pressures might facilitate earlier lung aeration without substantially increasing lung injury. Currently, uncertainty persists regarding the optimal initial CPAP strategy during neonatal transition [5].

From a physiological perspective, the primary objective of respiratory support immediately after birth is to facilitate clearance of foetal lung fluid, establish adequate FRC and achieve homogeneous lung aeration while preserving spontaneous breathing. Hooper and colleagues [9] described neonatal respiratory transition as a dynamic process in which successful lung aeration is essential for effective gas exchange and cardiovascular adaptation. Likewise, Dekker et al. [10] emphasised that maintaining spontaneous breathing during stabilisation enhances lung liquid clearance and promotes a more physiological transition, potentially reducing the need for invasive ventilation. Accordingly, the population in our study was intentionally restricted to infants born at 26 to 29 weeks’ gestation because they are more likely to establish spontaneous breathing immediately after birth, while remaining at high risk of surfactant deficiency. This way, they represent an appropriate population in whom to evaluate different CPAP strategies, thereby minimising the confounding effect of prolonged PPV during stabilisation.

Experimental studies have provided further support for this concept. Using preterm lamb models, Tingay and colleagues [11, 12] demonstrated that gradual and individualised lung recruitment strategies are more lung-protective than sustained inflations and result in improved pulmonary mechanics with less regional lung injury. These studies provided the physiological rationale for evaluating higher or individualised CPAP levels during neonatal stabilisation, as in the POLAR trial [13].

Our findings are consistent with recent clinical studies evaluating physiology-based respiratory support in the DR. In the OpenCPAP-DR trial, Duman et al. [14] reported that infants initially managed with lower CPAP levels more frequently required pressure escalation to achieve target oxygen saturation, whereas higher initial CPAP reduced the need for subsequent pressure adjustments without increasing major complications. Similarly, Martherus et al. [15] demonstrated the feasibility of a physiology-based CPAP strategy incorporating initially higher distending pressures, reporting improved heart rate stabilisation and shorter mask ventilation despite no significant differences in major respiratory outcomes. Mukerji et al. [16] also showed that higher CPAP levels may improve lung mechanics and reduce respiratory effort without significant haemodynamic compromise.

Interestingly, in our study, although the overall proportion of infants receiving surfactant did not differ between groups, infants receiving higher initial CPAP reached the predefined FiO_2_ threshold later than those allocated to lower CPAP. Several explanations may account for this finding. Earlier establishment of FRC may transiently reduce oxygen requirements despite persistent surfactant deficiency. Conversely, higher distending pressure may mask the physiological severity of lung disease when oxygenation alone is used to guide treatment decisions.

These observations also raise the possibility that physiological bedside assessment of lung aeration, for example by lung ultrasound, may complement oxygenation-based criteria when surfactant administration is considered. However, this hypothesis was not directly evaluated in the present study and warrants prospective investigation.

Our findings may also be interpreted within the evolving framework of precision respiratory medicine in neonatology [17]. Rather than considering CPAP as a fixed intervention, increasing evidence suggests that respiratory support during the immediate postnatal transition should be individualised according to the infant’s physiological response. De Luca and colleagues proposed that management of respiratory distress syndrome should integrate information on lung mechanics, surfactant deficiency and dynamic changes in pulmonary physiology rather than relying solely on predefined oxygen thresholds [2, 18]. In this perspective, the initial level of CPAP should probably be regarded as one component of a broader individualised recruitment strategy rather than as an isolated therapeutic target. Although our study was not designed to evaluate precision respiratory support, the different timing of surfactant threshold achievement observed between the two groups further highlights the need for physiological bedside tools capable of directly assessing lung aeration.

The strengths of this study include its randomised design, standardised DR management, continuous physiological monitoring using respiratory function monitoring, complete follow-up with no missing outcome data and rigorous protocol adherence. Furthermore, unlike many previous physiological studies, respiratory support was evaluated under routine clinical conditions, increasing the external validity of the findings.

This study has several limitations. First, as a pilot randomised trial, it was not powered to detect differences in clinically important respiratory outcomes or uncommon adverse events. Second, it was conducted at a single tertiary centre, potentially limiting generalisability. Third, blinding of the treating clinicians was not feasible because of the nature of the intervention. Finally, exploratory physiological outcomes should not be interpreted as evidence of treatment efficacy but rather as hypothesis-generating observations.

Future adequately powered multicentre randomised trials should evaluate individualised DR respiratory support strategies integrating physiological bedside monitoring, including lung ultrasound and other measures of lung aeration, to better define the optimal initial CPAP strategy during neonatal transition.

Ultimately, future research should determine whether optimising respiratory transition depends less on identifying a universally optimal CPAP level and more on tailoring distending pressure to each infant’s individual physiology.

## Conclusion

This pilot RCT demonstrated that comparing initial CPAP levels of 5 and 8 cmH_₂_O during DR stabilisation of very preterm infants is feasible and was not associated with major short-term safety concerns. Exploratory physiological findings suggest that higher initial CPAP may influence early respiratory adaptation and the timing of surfactant administration, although these observations should be considered hypothesis-generating. Larger adequately powered multicentre trials incorporating physiological assessment of lung aeration are required before changes in clinical practice can be recommended.

## Data Availability

Data will be provided upon reasonable request.
