# Correction to: Initial CPAP of 5 versus 8 cmH2O during delivery room stabilisation of very preterm infants: the PHILODENDROOM pilot randomised trial

**DOI:** 10.1007/s00431-026-07381-9

**Published:** 2026-09-03

**Authors:** Francesco Cavigioli, Ilia Bresesti, Francesca Castoldi, Sara Gatto, Enrica Lupo, Samantha Rossi, Azzurra La Verde, Silvia Bianchi, Fabio Meneghin, Francesca Viaroli, Valentina Pivetti, Valeria Manfredini, Giulia Cannata, Petrina Bastrenta, Paola Fontana, Ilaria Stucchi, Irene Daniele, Marco Chiera, Gianluca Lista

**Affiliations:** 1 NICU “V. Buzzi” Children’s Hospital, ASST FBF-Sacco, Milan, Italy; 2 Department of Medicine and Surgery, University of Insubria, Varese, Italy; 3 Foundation Centre, Osteopathic Medicine (COME) Collaboration, 65121 Pescara, Italy

**Correction to: European Journal of Pediatrics (2026) 185:673**

**https://doi.org/10.1007/s00431-026-07322-6**

In the article "Initial CPAP of 5 versus 8 cmH2O during delivery room stabilisation of very preterm infants: the PHILODENDROOM pilot randomised trial”, the name of one of the authors was incorrectly published.

• Published form: Irene Pivetti

• Corrected form: Valentina Pivetti

The original article has been corrected.

